# Gene Teller: an extensible Alexa Skill for gene-relevant databases

**DOI:** 10.1093/bioinformatics/btaa659

**Published:** 2020-08-21

**Authors:** Jon D Hill

**Affiliations:** Global Computational Biology and Digital Sciences, Boehringer Ingelheim Pharmaceuticals, Inc., Ridgefield, CT 06877, USA

## Abstract

**Summary:**

Voice assistants have become increasingly embedded in consumer electronics, as the quality of their interaction improves and the cost of hardware continues to drop. Despite their ubiquity, these assistants remain underutilized as a means of accessing biological research data. Gene Teller is a voice assistant service based on the Alexa Skills Kit and Amazon Lambda functions that enables scientists to query for gene-centric information in an intuitive manner. It includes several features, such as synonym disambiguation and short-term memory, that enable a natural conversational interaction, and is extensible to include new resources. The underlying architecture, based on Simple Storage Service and Amazon Web Services Lambda, is cost efficient and scalable.

**Availability and implementation:**

A publicly accessible version of Gene Teller is available as an Alexa Skill from the Amazon Marketplace at https://www.amazon.com/dp/B08BRD8SS8. The source code is freely available on GitHub at https://github.com/solinvicta/geneTeller.

## 1 Introduction

The past few years have seen remarkable growth in the use of voice assistants for access of information in the business and home. Devices such as Google Home and Amazon Echo have decreased dramatically in cost and continued to expand the scope and ability to access a variety of information resources. These assistants offer a natural way to interact by voice, providing advantages over screen-driven methods for speed of entry, and accessibility to populations with limited vision.

At the same time, the public databases containing gene-centric information of interest to biologists and other researchers continue to expand and refine themselves; through large public efforts, it is now possible for any researcher with internet access to obtain information about a gene’s expression, relation to different diseases, and known biology instantly and without cost through a web browser. This information is critical to researchers both in establishing fundamental biology, as well as those hunting for new targets for drug discovery.

However, these two core improvements have happened largely on parallel tracks in isolation of each other, aside from applications in laboratories advanced by academic labs (Lubiane-Alves *et al.*, 2018) and startups such as Helix (Halford, 2017). To overcome this limitation, the Gene Teller skill has been developed to enable researchers to query gene-relevant information from public sources quickly and easily. Moreover, it provides an extensible template for future developers to provide access to voice assistants to their gene-centric database in a cost effective and highly scalable fashion.

## 2 Materials and methods

The underlying resources in included in Gene Teller are pre-processed by Lambda functions that are written in Python 3.8 and triggered on weekly update intervals using CloudWatch events to ensure that the data remains current. These functions retrieve data from remote resources and parse the data to provide JavaScript Object Notation (JSON) files, which are dictionaries, keyed by the canonical gene symbol from Entrez Gene (Sayers *et al*, 2020). When the primary identifier of the data source is not this gene symbol, a second resource may be used to key the resource appropriately. These JSON files are compressed and stored in Amazon Web Services and Simple Storage Service (S3) for future access. If a resource supports multiple species, such as Refseq, one JSON file will be created for each species. Retrieval of a record via gene symbol will return a single value or a sorted list of values which can be iterated over by the skill.

An additional Lambda function builds a synonym-mapping JSON dictionary. Built from the NCBI gene-information file, this will take a mention of a gene synonym that is different from the canonical symbol, and map it back to the correct identifiers. In the case of ambiguity, a list of multiple values may be returned which can be clarified by the user.

The second group of components is triggered upon interaction with the voice assistant. An interaction model was built within the Alexa Skills Kit that recognizes utterances that request information about a gene. Different slots are used to denote variables within the request, such as the gene name. This slot has been populated with a list of gene symbols and full gene names from NCBI. Due to limitations within the size of the allowed model, these are not comprehensive; rather, they are used to train Alexa to determine the format of a reasonable gene reference in a request.

Alexa’s natural language model parses the requested gene, species (if provided) and requested information from the utterance based on the interaction model, and passes these to a Lambda function to return the result. This function will pre-process the gene utterance, and pass it to the synonym-mapping to get the canonical gene symbol. This identifier is used to retrieve the relevant information, which is then conveyed by the Alexa to the user. Some additional logic in the function allows for multiple requests for information about the same gene without explicit specification of the gene name, and allows Gene Teller to make requests enabling gene disambiguation. By default, the species is assumed to be *Homo sapiens*, but this may be specified in the query as well.

If multiple values are returned from a particular resource, such as GeneRIFs, the function will return the most recent value that has been added to the data source to the user upon the first retrieval, but will retain additional values to be presented upon request. This strikes a balance between overwhelming the user with a lengthy response and overly truncating the data.

## 3 Results

As a first implementation of Gene Teller, data from Entrez Gene, Refseq summaries (Pruitt *et al*, 2007),  and GeneRIF were processed for human, mouse and rat, and the human-specific resources ProteinAtlas (Thul & Linkskog, 2018) and Genotype-Tissue Expression (GTEx Consortium, 2017) were also incorporated into the JSON resources. This provides users with access to a variety of commonly sought information about a gene, such as the full name, the expression pattern of the gene, associations with genetic diseases and the latest published biological findings. The different resources currently available in the Gene Teller skill are highlighted in Table 1.

**Table 1.** Example of data included in Gene Teller

Resource	Information used	Example query
Entrez Gene	Gene identifier, synonyms, Refseq summary	What is the gene symbol for Entrez Gene 100?
GeneRIF	Literature associations of gene and function	Tell me a fact about CCR2.
GTEX	RNA Tissue expression	Where is the maximum expression of CCR1?
ProteinAtlas	Tissue specificity, subcellular localization	What is the tissue specificity of TNF?

For a standard conversation with the Gene Teller skill, the user adds it to their account on their chosen Alexa-enabled device. The user then activates Alexa with the chosen wake word (such as ‘Alexa’), and loads Gene Teller skill. Then, they can make requests for information using one of several natural-language invocations around the topic of interest. Gene Teller is able to provide information to the user even with use of less-common aliases, although due to the training data it provides accurate results with identifiers and synonyms that resemble gene symbols (such as GPA1 and LOC100294336) rather than non-standard-English phrases (‘IZUMO1 receptor JUNO’). For many simple tasks, such as conversion of an Entrez Gene to a human-recognizable name, it can be more efficient as a query interface than to navigate to the correct website and type in the lookup.

### 3.1 Incorporation of new data

To incorporate a new data source into Gene Teller, several modifications must be made to the skill itself and underlying Lambda functions

1. A new Lambda function is created which provides the updated JSON data in S3. This function will need to reformat data to be keyed by gene symbol if necessary.
2. A new interaction (including slots for gene and species) is described in the Alexa skill which triggers the retrieval of the new information.
3. A new section is added to the core Lambda function for retrieval of data and formatting the response
